# Gene mapping in white spruce (*P. glauca*): QTL and association studies integrating population and expression data

**DOI:** 10.1186/1753-6561-5-S7-I6

**Published:** 2011-09-13

**Authors:** John MacKay, Brian Boyle, Walid El Kayal, Marie-Claire Namroud, Trevor Doerksen, Janice Cooke, Nathalie Isabel, Jean Beaulieu, Philippe Rigault, Paul Bicho, Jean Bousquet

**Affiliations:** 1 Université Laval, Arborea, Centre for Forest Research and Institute for Systems and Integrative Biology, Québec, Québec, Canada; 2 Université Laval, Arborea, Centre for Forest Research and Institute for Systems and Integrative Biology, Québec, Québec, Canada; 3 University of Alberta, Department of biological Sciences, Edmonton, Canada; 4 Natural Resources Canada, Laurentian Forestry Center and Canadian Wood Fibre Centre, Québec, Québec, Canada; 5 Natural Resources Canada, Laurentian Forestry Center, Québec, Québec, Canada; 6 Gydle Inc., Québec, Québec, Canada; 7 FPInnovations, Vancouver, British Columbia, Canada

## Background

Connecting phenotype with genotype is the basis for developing forest genetic applications such as marker assisted selection (MAS). Quantitative Trait Locus (QTL) mapping and genetic association mapping (or linkage disequilibrium (LD) are two major approaches to find genes that control phenotypes of interest in forest trees. Quantitative trait loci (QTL) and association mapping experiments in white spruce (*Picea glauca* [Moench] Voss) aimed to identify genes linked to or associated with growth, adaptation, and wood property traits.

Gene mapping in conifer trees presents us with specific challenges, including very large genome sizes, low level of linkage disequilibrium, and large effective size in breeding populations. Therefore, association mapping experiments have relied on testing candidate gene targets rather than genome-wide association scans. We have explored different approaches to utilize gene expression and SNP outlier data to identify candidate genes, to help to explain the findings of gene mapping experiments and provide a broader understanding of observed phenotypic variations.

## Results

### Growth and phenology

The genomic architecture of bud phenology and height growth was investigated by assessing QTLs across pedigrees, years, and environments (1).A total of 11 distinct QTLs for bud flush, 13 for bud set, and 10 for height growth were localized on a linkage map highly-enriched in gene markers. Nearly 50% of the QTLs were stable across environments and/or years and 20% were replicated between populations. The proportion of phenotypic variance explained by QTLs ranged from 3% to 22.2%, and QTLs accounted for up to 70% of trait variance. These outcomes were integrated with findings from studies aimed identifying local adaption genes and gene expression associated with bud formation.

A genome-wide scan of 534 SNPs localized in 345 expressed genes was used to detect genes putative linked to local adaptation (2). We identified 5.5% of genes as outliers with FST at the 95% confidence level, and 14% of genes as candidates for local adaptation with a Bayesian method. The list of candidate genes and outliers includes sequences which co-localized with the QTLs for bud phenology.

A bud set roadmap was constructed by comprehensive microarray and qRT-PCR analysis of dormancy transition in bud, stem, needle, and root tissues over a time course, under short and long days (3). Tissue expression profiles were used to identify genes expressed only or preferentially in developing buds, which we hypothesize to play a more prominent role in bud formation. A core group of genes likely involved in the initiation of bud formation included about 100 of the bud-prominent genes and several sequences encoding potential regulatory proteins. Several of the bud set roadmap genes including bud-prominent genes co-localized with QTLs for the time of bud set.

### Wood properties

Wood physical traits were assessed using SilviScan technology in a population of 1700 trees comprising 215 open-pollinated families. In a pilot study, we tested for associations between single nucleotide polymorphisms (SNP) in 550 candidate genes and wood traits (4). We found 13 SNPs significantly associated with wood traits. The phenotypic variance explained reached up to 11% with approaches combining several SNPs.

Most association studies of wood properties have tested candidate genes that are highly expressed in secondary xylem, hypothesizing that genes that are preferentially or strongly expressed during wood formation are more likely to control wood properties. However, this hypothesis had not been tested. The genotyped sequences included genes with diverse expression profiles. Their transcript accumulation profiles were determined in trees grown under controlled conditions with a large-scale custom oligonucleotide microarray representing 25,094 different spruce genes. Of the 550 genes tested for association, 29% accumulated preferentially in secondary xylem compared to both secondary phloem and needles, but as many genes (29%) were phloem preferential. Xylem-preferential RNA accumulation was found for 10 of the 13 genes harbouring SNPs significantly associated. Our findings confirm that expression data were relevant for selecting candidate genes but not all of the genes containing significant SNPs were xylem preferential.

Transcript accumulation was also studied in secondary xylem of trees from the provenance-progeny trial, to further characterize the genes containing SNPs significantly associated with wood traits. In some cases, significantly different transcript levels were found among the different SNP genotypes. Xylem-preferential RNA accumulation was shown for the majority of these genes, which indicates that. Our results suggest that differential expression may be associated with SNP genotypes.

### Large-scale genotyping

A meta-analysis was used to integrate data from multiple experiments in order to identify and assign priorities to approximately 5000 candidate genes for association mapping experiments. The candidate gene selection considered the above gene expression data, findings from transcriptomic investigations of gene regulation, studies investigating transcriptional variation within mapping populations trees, and outlier data related to local adaptation. A large-scale genotyping chip was developed and data were obtained for 7000 SNPs from nearly 2500 genes.

## Conclusions

This report describes QTL mapping and genetic association mapping results. We have illustrated ways in which gene expression and population data may be of value in these approaches, whether they are used to select candidate genes or to characterize the physiological processes underlying marker-trait associations.
